# Retrospective temporal resolution interpolation alters myocardial strain quantification on compressed sensing cine CMR

**DOI:** 10.1007/s10554-025-03348-3

**Published:** 2025-02-14

**Authors:** Leonard Grob, Yann Schwerzmann, Dario Kaiser, Bernd Jung, Thilo Schweizer, Stefan P. Huettenmoser, Scilla Dozio, Adrian T. Huber, Martina Boscolo Berto, Christoph Gräni, Dominik P. Guensch, Kady Fischer

**Affiliations:** 1 Department of Anaesthesiology and Pain Medicine, Bern University Hospital, Inselspital, University of Bern, Freiburgstrasse 10, Bern, 3010 Switzerland; 2 Department of Diagnostic, Interventional and Paediatric Radiology, Bern University Hospital, Inselspital, University of Bern, Bern, Switzerland; 3 Translation Imaging Center (TIC), Swiss Institute for Translational and Entrepreneurial Medicine, Bern, Switzerland; 4 Department of Radiology and Nuclear Medicine, Lucerne Cantonal Hospital, University of Lucerne, Lucerne, Switzerland; 5 Department of Cardiology, Bern University Hospital, Inselspital, University of Bern, Bern, Switzerland

**Keywords:** Cardiovascular magnetic resonance, Compressed sensing, Myocardial strain, Cardiac function, Retrospective temporal resolution interpolation

## Abstract

Compressed sensing (CS) is a rapidly developing technique that can acquire functional cines of the heart within seconds while free-breathing and it is ideal for assessing cardiac function in non-typical conditions such as when patients are sedated or anaesthetized or undergoing stress exams. CS cines can further include retrospective temporal resolution interpolation (INTP_TR_) to improve the frame count per heartbeat, and the impact of INTP_TR_ on biventricular functional measurements is unknown. We investigated the impact of INTP_TR_ on left and right ventricular volumetry and strain measurements of CS cines. Nineteen patients with 51 different CS acquisitions were prospectively enrolled. CS cines were acquired at rest, under adenosine stress, oxygen inhalation or while under general anaesthesia with mechanical ventilation. From the same CS acquisition, a dataset with and without INTP_TR_ were generated by the scanner. The outputs were separated and analysed by blinded readers for left and right ventricular volumetry, as well as systolic and diastolic strain parameters using feature-tracking techniques. Measurements were compared between the INTP_TR_ and non-INTP_TR_ outputs. Similar measurements were obtained for biventricular volumes and ejection fraction independent of INTP_TR_. Peak strain was significantly underestimated on INTP_TR_ cines for both longitudinal and circumferential orientations (*p* < 0.01). Nevertheless, good-to-excellent correlations were observed between the two measurements (*r* > 0.65, *p* < 0.01), and there was still a high area under the curve (AUC ≥ 0.95, *p* < 0.01) for detecting abnormal patients defined by strain analysis on the standard segmented cine. INTP_TR_ especially negatively influenced strain rates analysis, as many strain rate curves were deemed unusable with this technique. These findings were consistent independent if the patient was in a resting, stress or anaesthetized condition. Although INTP_TR_ is a feature which improves temporal resolution on CS cines, quantification of biventricular strain and strain rates is not feasible or comparable, thus, feature tracking analysis should be performed on non-INTP_TR_ data. However, volumetry and ejection fraction analysis are consistent independent of which output is analysed.

**Supplementary Information:**

The online version contains supplementary material available at 10.1007/s10554-025-03348-3.

## Introduction

Cardiovascular magnetic resonance (CMR) is a key imaging technique in the assessment of cardiovascular disease, however, one of its limitations had been the extended scan time and need for breath-holds to acquire traditional segmented cines. This is not ideal for exams where rapid scanning is required or for patients where longer breath holds may not be feasible [1]. In these cases, image quality is reduced or alternative imaging modalities are used. Therefore, compressed sensing (CS) techniques have emerged to accelerate image acquisition [2]. These obtain comprehensive images of the entire heart in seconds while the patient can breathe freely [3–5]. Application of free-breathing CS imaging has been shown to be useful in stress exams, for paediatric patients who may not be able to follow breathing commands or may need sedation during MR-scans [6–8], and for patients under general anaesthesia who are mechanically ventilated where breath-holds have to be manually performed by turning off the ventilator [9, 10]. CS cines have already been validated against traditional segmented cines for quantification of standard clinical parameters such as right and left ventricular volumes and ejection fraction (EF) [11–14]. While EF is the most common and validated marker, it can be confounded by geometry of the heart [15], leaving myocardial deformation assessment, or strain, an ideal option for quantification of cardiac function. With CMR, atrial and ventricular strain can be quantified on any cine using post-processing techniques such as feature tracking (FT) [16, 17]. In the last five years, this analysis has also been extended to investigate if FT analysis of CS cines can provide reliable results [18].

As CS imaging continually develops, sequences now may include retrospective temporal resolution interpolation (INTP_TR_). For example, a typical product CS cine using parameters provided by the manufacturer may output a dataset with approximately 45ms between images. Using INTP_TR_ a second dataset is output from the same acquisition based on the number of cardiac phases defined by the user. For example, programming a CS cine to have 30 phases per cardiac cycle typically results in a cardiac phase every 25–30 ms for a heart rate of 60-70 bpm. To obtain this increased frame rate or better temporal resolution (TR), characterized by a shorter TR duration, the INTP_TR_ cines are retrospectively reconstructed out of the original CS cines and do not include the original image. A shorter TR should theoretically lead to better assessment of the cardiac motion. It has been demonstrated that improved TR on standard breath-hold cines leads to higher LV global circumferential and radial strain values [19] and higher left atrial strain rate [20, 21]. However it is unknown if an increase in frames by INTP_TR_ has the same effect on CS cines. Thus, the purpose of this study was to investigate the impact of INTP_TR_ on quantification of volumetric and strain analysis of CS CMR cines. A secondary aim was also to investigate this comparison during differing conditions of image acquisition including a normal resting state, during stimuli (adenosine stress and oxygen inhalation) or while under general anaesthesia.

## Methods

### Dataset inclusion

In total, 51 CS acquisitions were considered for analysis from healthy controls (*n* = 18 acquisitions, *n* = 6 participants) and patients with coronary artery disease (CAD, *n* = 33 acquisitions, *n* = 13 patients). Participants were prospectively recruited to undergo a CMR exam for research purposes and provided written informed consent for secondary use of their data. This study was approved by the Cantonal Research Ethics Board of Bern, Switzerland (#2020 − 01258) and complies with the Declaration of Helsinki.

### CMR image acquisition

Patients underwent a CMR exam in a 3.0-Tesla clinical scanner using the dedicated 18-channel body coil with a 72-channel spinal coil (Siemens Magnetom Vida, Siemens Healthineers). CS cine images were acquired in three long axis (LAX) views and as a short axis (SAX) stack covering the entire left (LV) and right ventricle (RV). Typical parameters for the CS were: Balanced steady state free procession sequence, image matrix of 170 × 208, reconstructed voxel size of 2.0 × 2.0mm^2^ for the SAX and 1.7 × 1.7mm^2^ for the LAX, slice thickness of 6.0 mm, flip angle of 60°, 16 segments, bandwidth of 859 Hz/Px echo time of 2.23ms and an original repetition time of 45ms interpolated for 30 phases per cardiac cycle (i.e. for an RR interval of 1000ms, INTP_TR_ was reconstructed to 33ms). For each CS acquisition two datasets were output, (1) the non-INTP_TR_ with a repetition time of 45ms (i.e. 22 phases for an RR-interval of 1000ms), and (2) the INTP_TR_ with 30 phases per cardiac cycle to match the clinical segmented cine in place at the local institute. In all participants CS images were acquired at an awake baseline state while free-breathing, and a second CS dataset was acquired during an end-expiration breath-hold. To compare INTP_TR_ and non-INTP_TR_ in a non-resting state, participants either remained awake during the exam and images were reacquired under two individual stimuli (supplemental oxygen, 10 L/min with a facemask, or adenosine infusion of 140 µg/kg/min), or patients underwent scheduled induction for general anaesthesia with mechanical ventilation and images were reacquired ten minutes into anaesthesia maintenance [9].

Additionally, in either a baseline resting state or while breathing supplemental oxygen, standard segmented cines used in typical CMR clinical exams were acquired during end-expiration breath-holds in SAX and LAX views. Typical parameters for the segmented cine were: Retrospective ECG gated balanced steady state free procession sequence, image matrix of 174 × 208, reconstructed voxel size of 1.7 × 1.7mm^2^ for the LAX, slice thickness of 8.0 mm, bandwidth of 962 Hz/Px, flip angle of 45°, echo time of 1.40ms, 11 segments and 30 phases per cardiac cycle.

### CMR image analysis

All CMR images were re-coded to blind readers from the patient information. Analysis was performed with Circle Cardiovascular Imaging (version 5.17, Calgary, Canada). For each compressed sensing acquisition, the non-INTP_TR_ and INTP_TR_ were analysed separately for standard volumetric and anatomical measures and for FT analysis. Based on the heart rate at the time of acquisition, different phase numbers may have been present between slices for non-INTP_RT_ datasets. If this occurred, due to requirements of the software, slices were adapted to an identical phase count by removing phases at the end of the cardiac cycle if the reader visually determined no cardiac movement in these removed images. If this was not the case, slices were analysed separately. In INTP_TR_ images, analysis of all slices was performed simultaneously due to an equal phase number (30 phases).

Volumes of both the RV and LV, along with LV mass were analysed, following standard techniques using automatic epicardial and endocardial contour detection at end diastole and end systole on SAX cine stacks. Contours were adjusted manually if needed. For FT analysis, automatic end-diastolic and end-systolic contours were placed on the end-diastolic frame for the LAX and SAX-stack. In the latter, slices incorporating the outflow tract were excluded. If required, additional contours were placed throughout the cardiac cycle to improve tracking. Global peak strain and systolic, as well as early and late diastolic strain rates were assessed for circumferential and longitudinal strain [17, 22]. If the reader determined the tracking or measures to be unreliable (based on the corresponding strain and strain rate curves), the parameter was excluded. Acceptable tracking was defined by the reader if the software contours and mesh model followed the endocardial and epicardial borders throughout the entire cardiac cycle. The strain and strain rate curves were then judged by the reader, and if they were determined to not be reliable and in line with published curves [22], for example too many oscillations where peaks could not be detected by the reader, then the measurements were excluded.

### Statistical analysis

Data are reported as mean ± SD. First the percentage of parameters for each analysis included by the readers was reported. If at least 10 datasets were available, then comparisons between non-INTP_TR_ and INTP_TR_ measures were assessed with a paired t-test and a Pearson’s correlation. For resting datasets with a corresponding segmented cine, a repeated measures ANOVA was used to compare the three measures, if more than four matching datasets were available. Finally, to assess if the difference in TR contributed to the discrepancy between non-INTP_TR_ and INTP_TR_, a non-linear regression compared the absolute difference in ventricular measures to the difference in TR. Secondary analysis then compared non-INTP_TR_ and INTP_TR_ measures for the individual conditions; (1) during end-expiration breath-hold in resting conditions, (2) while free-breathing in resting conditions, (3) during free breathing under a short-acting stimulus (adenosine or supplemental oxygen, face mask at 10 L/min for 5 min), or (4) while under general anaesthesia with respiration controlled by positive pressure ventilation using a one-way ANOVA. Statistical significance was defined with a two-sided *p* value of < 0.05. GraphPad Prism version 10 (GraphPad Software, La Jolla California USA) was used for statistical analysis.

## Results

### Participant characteristics and data inclusion

The median age was 48 years (range 18–77). Left ventricular ejection fraction was > 40% for all datasets, and no patients had dilated or hypertrophic hearts (supplemental Table 1).

**Fig. 1:**
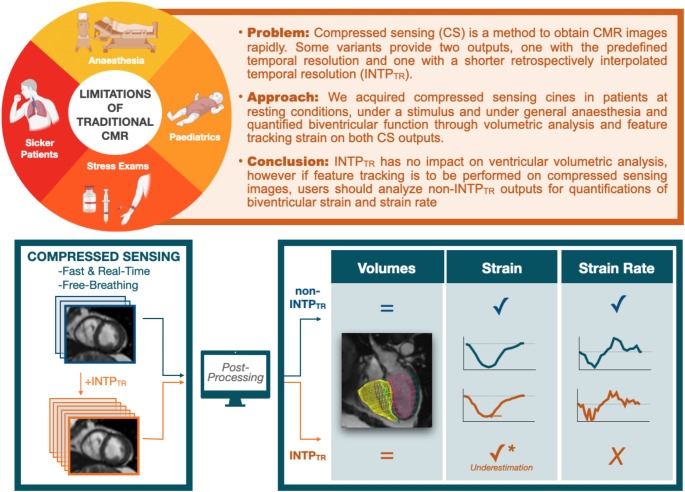
Take home figure. CMR: cardiovascular magnetic resonance

**Fig. 2:**
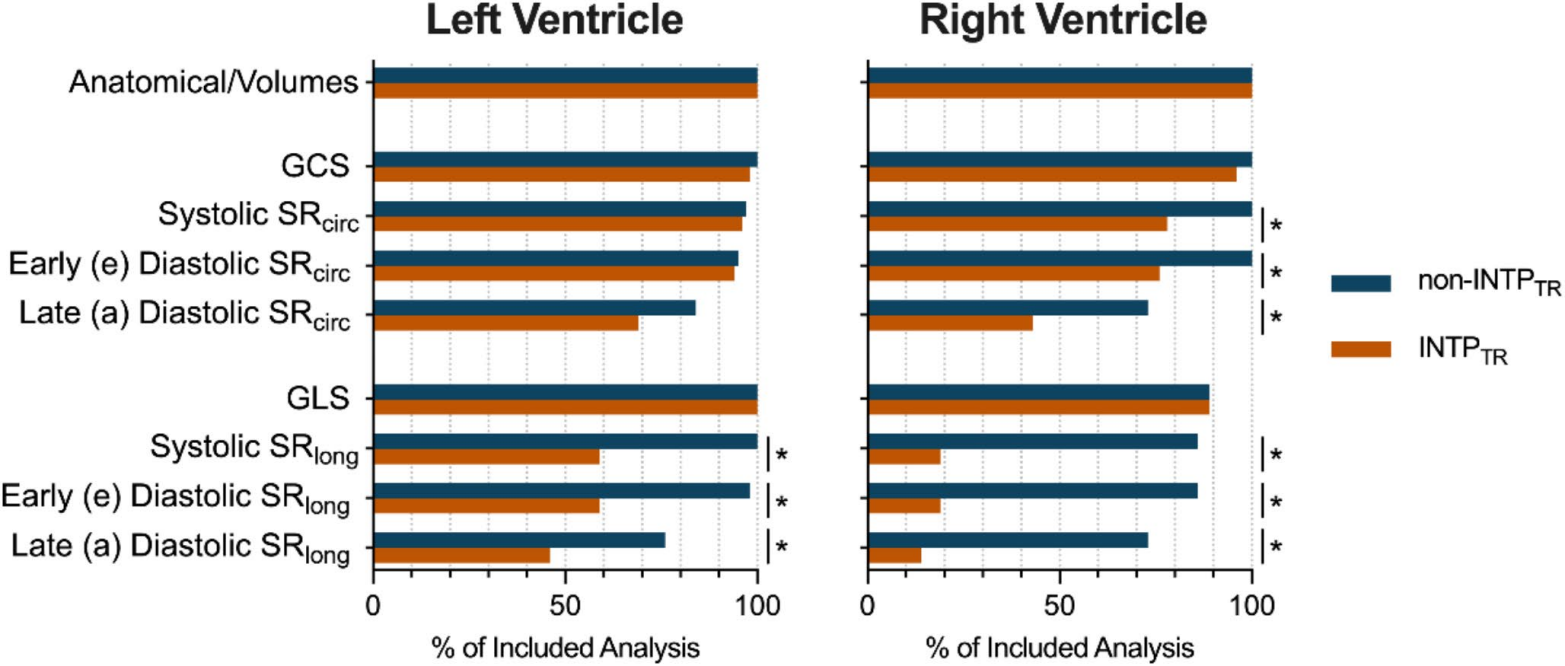
Inclusion of quantitative measurements. Direct comparison of inclusion rates in percentage between the retrospective temporal resolution interpolation (INTP_TR_, orange) and the non-INTP_TR_ (blue) sequence. Volumetric measures and peak strain measures show high inclusion rates of over 90%, while especially for longitudinal strain rates, inclusion rates were significantly lower with the INTP_TR_ datasets (*p < 0.05). GCS: global peak circumferential strain, GLS: global peak longitudinal strain, SR: strain rate

In total, from the 51 datasets acquired, SAX CS cines were obtained in *n* = 49 (awake baseline state (*n* = 26), during a stimulus (oxygen inhalation or adenosine infusion, *n* = 14), while under general anaesthesia (*n* = 9)), while LAX CS cines were obtained in *n* = 37 (awake baseline state (*n* = 28), while under general anaesthesia (*n* = 9)). Standard anatomical and volumetric assessments of both ventricles could be performed for all acquired datasets (Figs. 1 and 2). For LV feature tracking analysis, the myocardial tracking was deemed acceptable for 100% of the SAX and LAX CS cines, and peak strain (GCS and GLS) was acquired in all non-INTP_TR_ and INTP_TR_ datasets. In the RV, peak strain in both orientations was acquired from at least 89% of the data, with no difference between the non-INTP_TR_ and INTP_TR_. However, with strain rates, there was differentiation in inclusion between INTP_TR_ and non-INTP_TR_. Despite acceptable tracking, peak systolic and diastolic strain rates waves, especially in the longitudinal orientation, could not be as reliably determined from the INTP_TR_ strain rate curves while many systolic and early diastolic strain rates were still defined by the reader with non-INTP_TR_. The differences in the calculated strain rate curves can be clearly observed in Fig. 1. In particular, strain rates in the longitudinal direction of the RV were often excluded by the reader with the INTP_TR_ datasets, resulting in less than 20% inclusion.

### Comparison between INTP_TR_ and non-INTP_TR_

There was no difference in either LV or RV volumes, mass or ejection fraction between the INTP_TR_ and the non-INTP_TR_ quantifications (Supplemental Table 1). Specifically for common LV functional measurements, ejection fraction was 56 ± 5% with the INTP_TR_ and 57 ± 5% for the non-INTP_TR_ (*p* = 0.30), and cardiac index was also the same (2.9 ± 0.7 L/min/m^2^ vs. 2.9 ± 0.7 L/min/m^2^, *p* = 0.30). Furthermore, all biventricular volumetric measures were highly correlated between the INTP_TR_ and the non-INTP_TR_ CS cines (Supplemental Table 3).

**Fig. 3:**
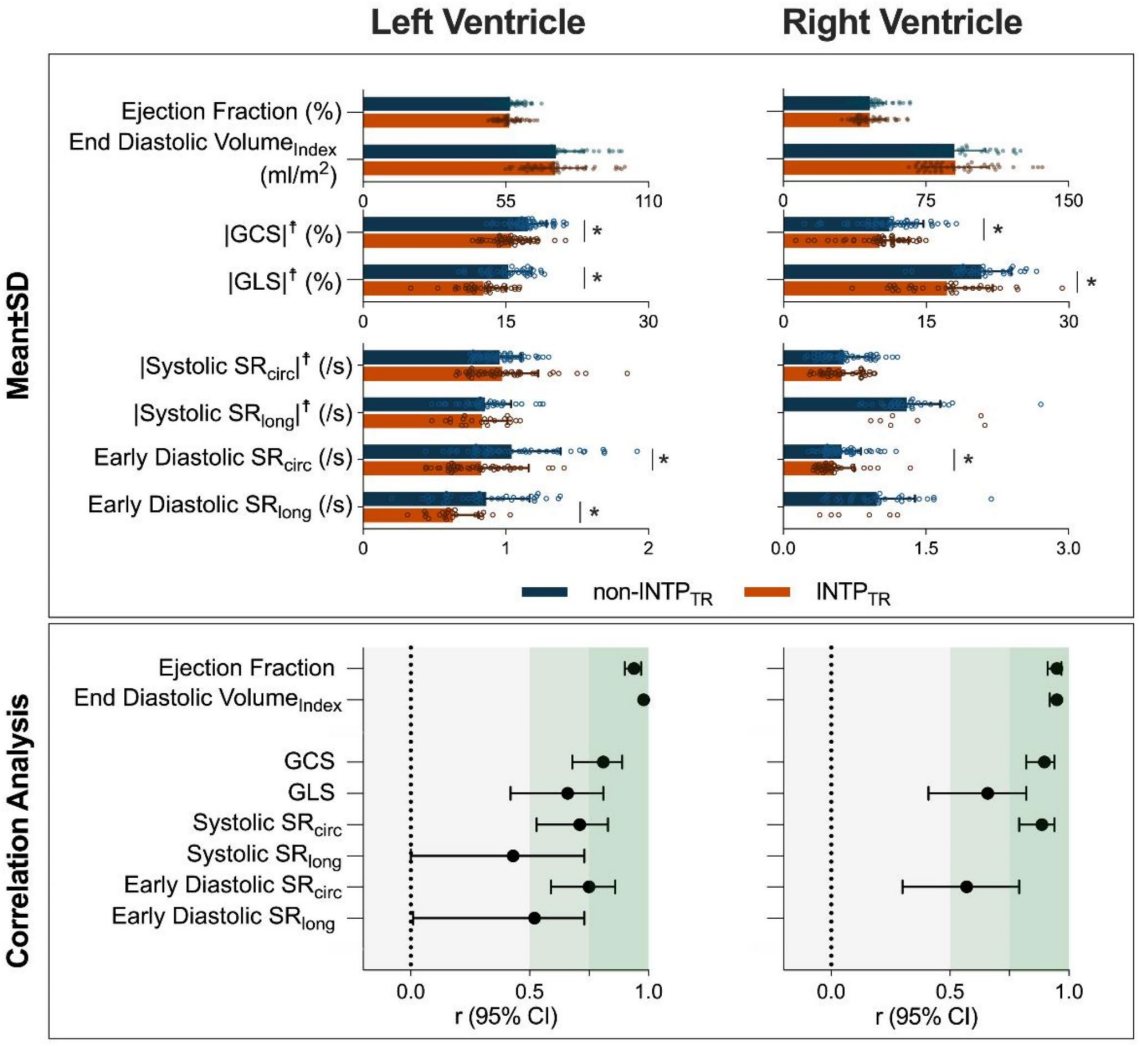
Comparison of biventricular measurements on compressed sensing cines with and without retrospective interpolated temporal resolution (INTP_TR_). Top: Group analysis (mean ± SD) showed that while standard volumetric measures did not differ between compressed sensing cines without (blue) and with INTP_TR_ (orange), feature tracking measurements showed that peak strain and diastolic strain rates (SR) were significantly lower with the INTP_TR_ output (*p < 0.05) for both circumferential (circ) and longitudinal (long) orientations. ☨ for display purposes |Absolute| values (i.e. multiplied by -1) are shown for the global peak strain and systolic strain rates. Bottom: Correlation analysis demonstrates excellent (dark green zone) correlation for volume assessments and good (light green zone) correlations for most feature tracking measurements except for longitudinal strain rate measurements. Statistical analysis of right ventricular strain rates was not performed due to high exclusion rates

In contrast to volumetry, significant differences were observed with feature tracking measurements. LV and RV peak strain were both lower with the INTP_TR_ analysis in comparison to the non-INTP_TR_ for GCS (LV: -15.6 ± 2.0% vs. -17.3 ± 2.0%, *p* < 0.01, RV: -10.2 ± 3.0% vs. -11.2 ± 3.5%, *p* < 0.01) and GLS (LV: -12.5 ± 2.4% vs. -15.3 ± 2.4%, *p* < 0.01, RV: -17.2 ± 4.8% vs. -20.8 ± 3.1%, *p* < 0.01) in comparison to the non-INTP_TR_ output. Despite the disagreement, excellent correlations (LV: *r* = 0.81, *p* < 0.01, RV: *r* = 0.90, *p* < 0.01, Fig. 3) were observed between the datasets in a circumferential orientation, and good correlations for longitudinal orientation (LV: *r* = 0.66, *p* < 0.01, RV: *r* = 0.66, *p* < 0.01). Systolic strain rate did not differ, but weaker correlations were observed, especially for the longitudinal orientation, in comparison to peak strain and volumetric values. Similar to peak strain, early diastolic strain rates were underestimated with the INTP_TR_. Because so few late diastolic peaks (a-wave) were observed with the INTP_TR_, no further statistical analysis was performed with this marker, nor were enough data points available for RV longitudinal strain rates for any time point.

### Impact of the TR ratio

The average TR from the INTP_TR_ was 29 ± 6ms, which was dependent on the RR interval at the individual acquisition. In comparison to the set TR of 45ms of the non-INTP_TR_, the mean TR-ratio was 1.6 ± 0.3, indicating the interpolated TR was approximately 1.6-fold shorter than the acquired TR. The range in TR ratio was from 1.0, indicating both outputs had a similar TR, to 2.4 where in some acquisitions INTP_TR_ was only 19ms. Correlation analysis (Supplemental Table 4) demonstrated that the difference in peak strain measurements was not dependent on the TR-ratio. However, many of the systolic and early diastolic strain rate markers were significantly correlated to the TR-ratio, where if there was a larger difference in the TR-ratio, then the INTP_TR_ had a greater underestimation of strain rates in comparison to the non-INTP_TR_.

### Influence of the patient condition at acquisition

**Fig. 4:**
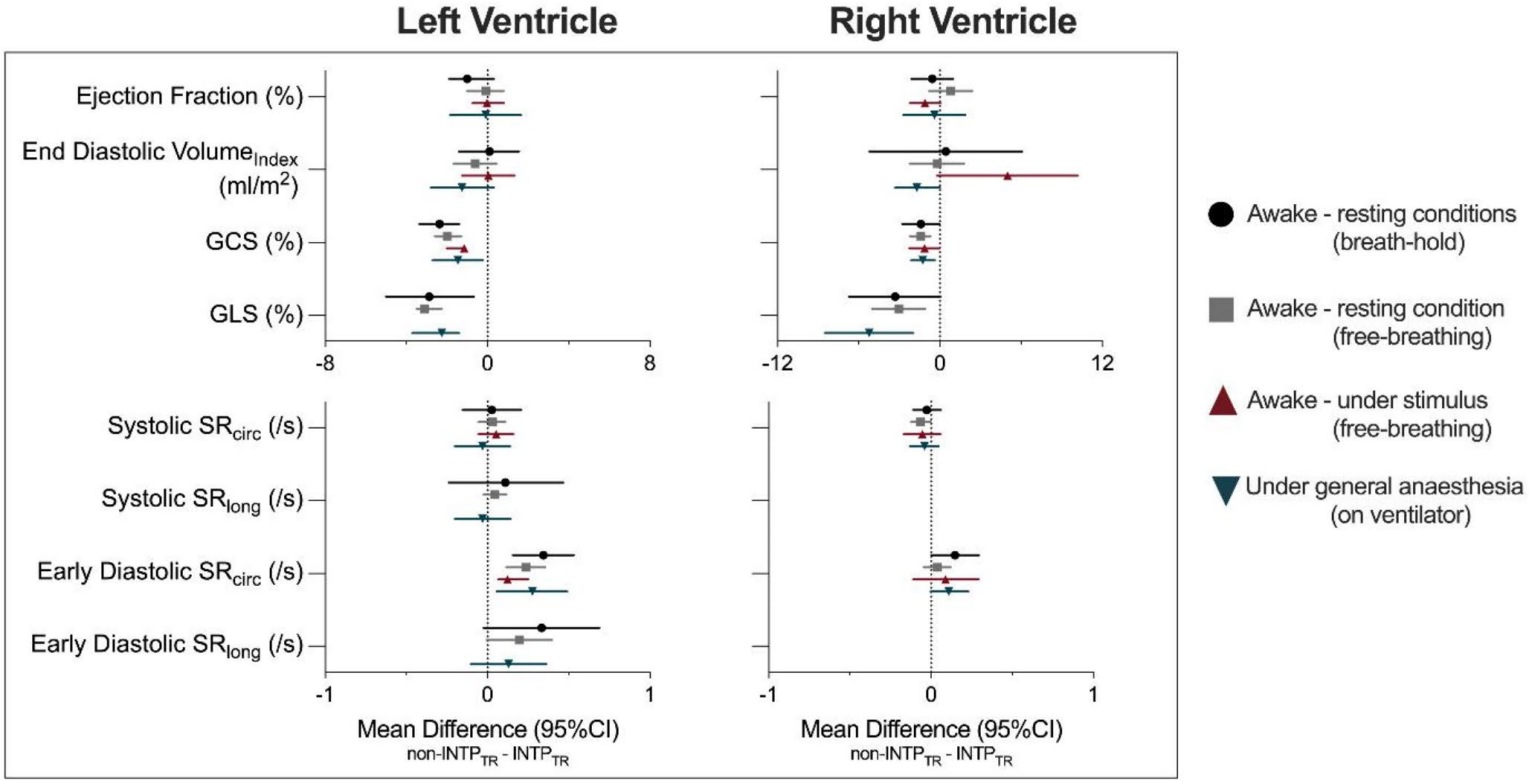
Patient status does not impact the discrepancies between INTP_TR_ and non-INTP_TR_ measurements. Mean differences (95% confidence intervals) of measurements between compressed sensing cines with (INTP_TR_) and without (non-INTP_TR_) retrospective interpolated temporal resolution were the same, independent of the patient status during which the images were acquired. As peak strain and systolic strain rates are negative values, a negative mean difference indicates that measures are stronger with the non-INTP_TR_, while with ejection fraction, volumes and early diastolic strain rate a positive difference means that measures are higher with the non-INTP_TR_. Statistical analysis of right ventricular strain rates was not performed due to high exclusion rates

As shown in Fig. 4, the conditions under which the images were acquired did not significantly impact the differences observed between INTP_TR_ and non-INTP_TR_ measurements. The same trends were observed if the patient was awake and images were acquired during a breath-hold or while free-breathing, if images were acquired in an awake patient during a stimulus (adenosine or supplemental oxygen), or if the patient was anaesthetized with breathing regulated by a ventilator.

### Comparison to segmented breath hold cines as the gold standard

**Fig. 5:**
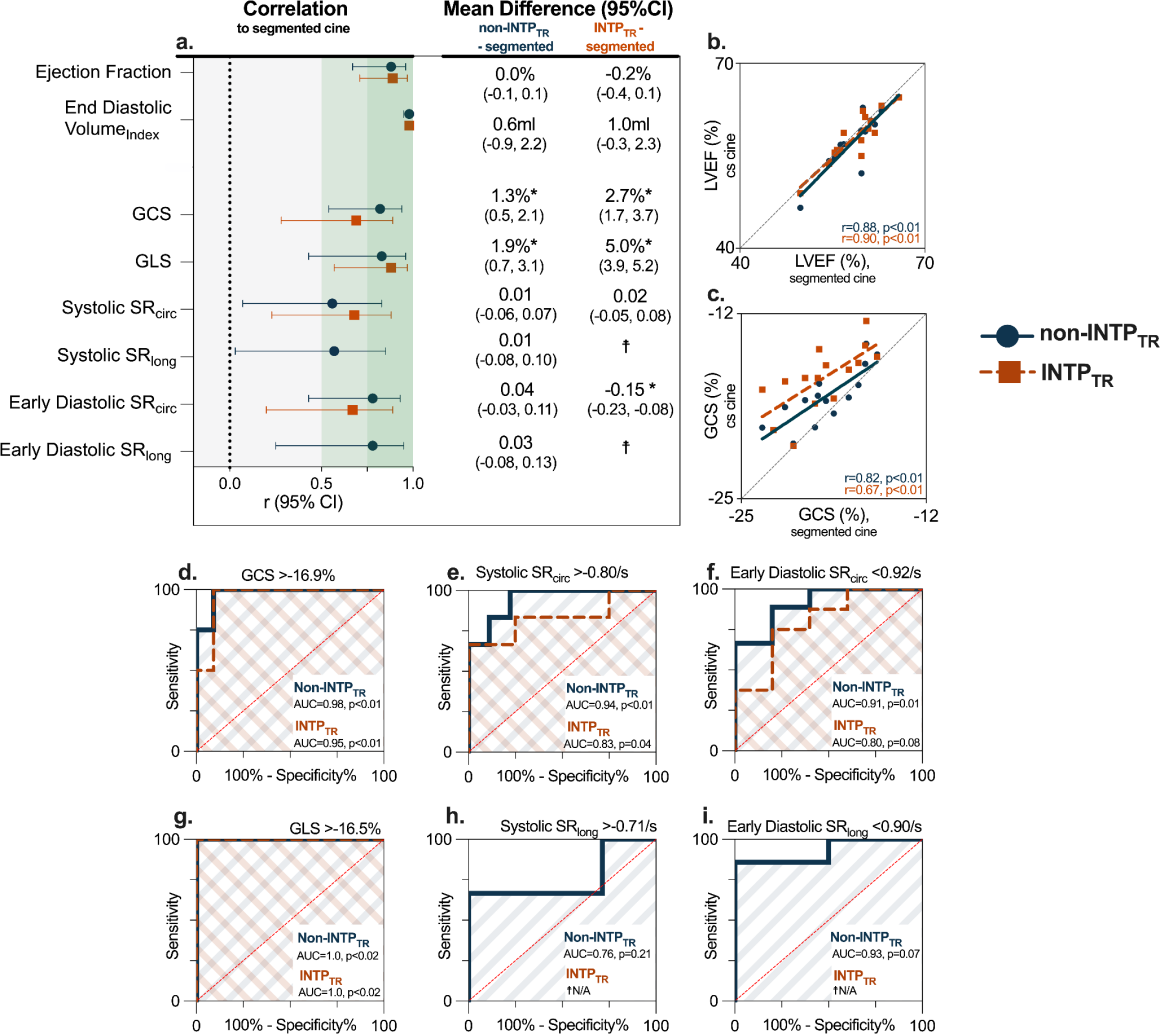
Comparison of compressed sensing measurements to standard segmented cines. (**a**) Correlation coefficients and mean differences are displayed between the measurements derived from compressed sensing cines without (blue) and with (orange) INTP_TR_ in comparison to the analysis acquired from standard segmented cines. (**b**) correlation graphs of LVEF depict high correlations right on the line of identity demonstrating no bias between measurements, in comparison to the GCS correlation graphs (panel **c**), where there are high correlations but analysis from the non-INTP_TR_ and INTP_TR_ underestimate peak strain. Nevertheless, both compressed sensing techniques can detect abnormal patients (defined by the segmented cine strain analysis) with a high accuracy for GCS (**d**). Additionally systolic (**e**) and early diastolic (**f**) strain rate analysis from non-INTP_TR_ depicts similar measures, while lower AUC measures are obtained from the INTP_TR_ quantifications. AUC curves are shown for longitudinal strain measures in **g**–**i**). *p < 0.05 represents a significant difference from the compressed sensing analysis to the segmented cine analysis. ☨Measurements with less than *n* = 10 comparisons were not statistically compared. CS: compressed sensing, GCS: peak global circumferential strain, GLS: peak global longitudinal strain, LVEF: left-ventricular ejection fraction, SR: strain rate

In the same conditions, a limited sample of seventeen standard segmented cines were available for comparison to the CS analysis. There were no differences in ejection fraction nor end-diastolic volume from either the non-INTP_TR_ and INTP_TR_ analysis in comparison to the segmented cine analysis (Fig. 5), with excellent correlations observed for both LV ejection fraction (non-INTP_TR_*r* = 0.88, *p* < 0.01, INTP_TR_, *r* = 0.90, *p* < 0.01) and end diastolic volume index (non-INTP_TR,_*r* = 0.98, *p* < 0.01, INTP_TR_, *r* = 0.98, *p* < 0.01, RV values are provided in Supplemental Table 3). It can be observed that for strain measurements, the non-INTP_TR_ still underestimates biventricular GCS and GLS in comparison to the standard cine, while this underestimation is even greater with the INTP_TR_. Specifically for peak strains this underestimation was consistent, yielding good correlations between the measurements. This can be observed in the correlation panels of Fig. 5, where a good correlation is observed. Yet there is a shift from the line of identity indicating an underestimation of GCS from the analysis of the CS cines in comparison to the analysis of segmented cines, whereas this difference is not observed in the ejection fraction panel. Subsequent ROC statistical analysis showed the capabilities of both GCS and GLS (non-INTP_TR_ and INTP_TR_) to detect abnormal patients defined by the segmented breath-hold cine were still excellent (area under the curve (AUC) > 0.95 for all). Systolic and early diastolic strain rates did not differ for the non-INTP_TR_, and good to excellent correlations and AUC were observed. For the INTP_TR,_ slightly lower AUC were observed for circumferential systolic and early diastolic strain rates, while longitudinal strain rate analysis could not be performed due to a high exclusion rate (Supplemental Table 5).

## Discussion

In this investigation we compared CS cine acquisitions with and without INTP_TR_ and observed that the reconstruction with INTP_TR_ did not impact the quantification of biventricular volumes, but deformation analysis using feature tracking techniques was influenced by INTP_TR_.

CS techniques improve imaging workflow and represent the future of CMR, imaging rapidly without breath-holding. Thus, CS increases the likelihood of obtaining usable cardiac images in non-ideal or non-standard situations including stress exams or in patients where performing repetitive breath-holds is not feasible, such as during general anaesthesia and it is likely to be incorporated into more exams in the future as it can significantly decrease scan time [8]. To the best of our knowledge, while multiple groups have assessed how functional measurements of compressed sensing compare to standard segmented cines [4, 6, 7], it has not yet been published how ventricular measurements may be impacted by data outputs that have INTP_TR_ or not. Therefore, after choosing to implement compressed sensing, the subsequent important decision for clinicians and researchers is to decide on what output to analyse. Our findings show that for quantifying standard markers such as biventricular volumes and myocardial mass, the results do not differ whether analysis is performed on the INTP_TR_ or non-INTP_TR_ output. However, when quantifying strain using feature tracking, additional considerations are needed. Importantly for peak strain (GCS and GLS), a linear trend is observed between the non-INTP_TR_ and INTP_TR_ measurements and the standard segmented analysis, although different absolute values are obtained. This indicates that strain analysis can be performed on CS images, however, different reference values should be determined for either output. The largest discrepancies occurred with strain rate analysis. While strain rate analysis performed relatively well on the non-INTP_TR_ images, this was not the case for the INTP_TR_ images, where especially for the longitudinal orientation and the right ventricle, strain rates were excluded. Consequently, this data indicates that feature tracking strain is to be performed on CS images without INTP_TR_ and an adapted range of normal values may be needed.

### Ventricular volumes and mass

For the LV volumes and anatomy, multiple groups have shown that CS is equal or fairly comparable to the standard imaging [13, 23], while it is particularly advantageous when assessing biventricular function during continuous exercise at different difficulties [24]. Even with the complex geometry of RV function and volume in congenital heart disease, Longère et al. demonstrated that CS imaging provided equivalent findings to gold-standard segmented imaging, while significantly reducing scan time [25]. Nevertheless, there are some known limitations. Due to the prospective nature of CS imaging, it is possible that the end-diastolic image can be lost leading to slight underestimations of end-diastolic volume or mass [12]. However, these are often minimal differences, as shown by Ma et al., where LV mass differed by less than 2 g in comparison to the reference standard [23]. In our analysis we did not observe these differences with either the non-INTP_TR_ nor INTP_TR_, even under a stimulus where heart rate may be increased. Importantly, we observed equal agreement for both the left and the right ventricle, which is significant, as biventricular assessment is gaining momentum [26].

### Ventricular peak strain

With peak strain measurements there is discrepancy in the field if quantifications from the CS cines match the reference standard or not. It has been demonstrated that with free breathing CS imaging, strain tends to be underestimated compared to standard segmented imaging. This was shown to be mainly due to blurring in the endocardial edges [27] and not by different respiratory modes during acquisition [23, 28]. Li et al. [20] found significant underestimation in all three strain orientations with CS cines compared to segmented cines, while other research groups showed no significant difference in LV circumferential peak strain [12, 29]. In our results, although we see biventricular strain is underestimated both circumferentially and longitudinally, the difference is significantly larger with the INTP_TR_. Thus, it is likely that the differences are caused by variations within the CS cines, and for each variant used, a unique set of reference values may be required. Although we observed that peak strain was underestimated, the high AUC, strong correlations and lack of dependency on the TR ratio indicate both non-INTP_TR_ and INTP_TR_ CS could be used for comparisons when implementing the same sequence, i.e. comparing cardiac function at rest to cardiac function during a stimulus or anaesthesia [9, 30, 31]. It should be mentioned strain analysis is not limited to feature tracking, but other post-processing techniques are available for quantifying cardiac function. For example, Backhaus et al. successfully implemented real-time CMR to detect HFpEF during exercise stress using manual long axis strain of the LV and LA instead of deformation analysis [32].

### Ventricular strain rates

**Fig. 6:**
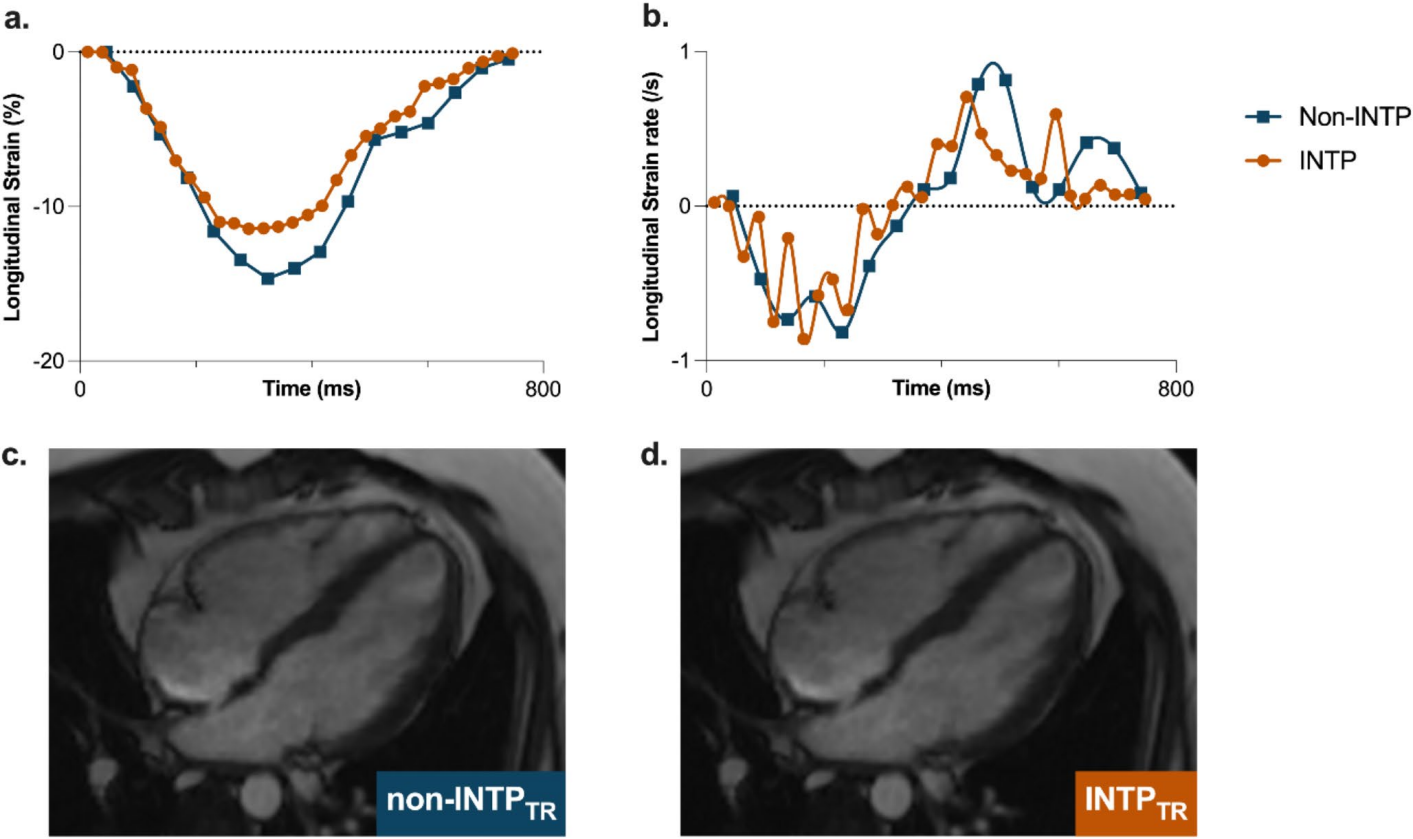
Case example. Panel **A** and **B**: Peak strain curves and strain rate curves are shown for the retrospective temporal resolution interpolation (INTP_TR_) and the non-INTP_TR_ sequence, originating from the same dataset. The orange lines show the INTP_TR_ strain and strain rate curves, and the blue lines show the non-INTP_TR_ curves. Specifically in the strain curves of the INTP_TR_, phase to phase fluctuations are visually apparent. Panel **C** and **D:** 4 chamber images with INTP_TR_ (panel **C**) and without INTP_TR_ (panel **D**) of the same dataset at the same time point in the cardiac cycle

The final category of functional assessments we quantified was strain rates. Strain rate analysis is particularly useful at assessing ventricular lusitropy and diastolic function. This is typically defined with the early (e-wave) and late (a-wave) peak diastolic strain rates [17]. For example, in a perioperative environment using echocardiography, diastolic strain rates have been used to assess intraoperative diastolic dysfunction in patients undergoing cardiac surgery [33]. Moreover, diastolic strain rates have been used with both echocardiography and CMR during stress tests to identify changes in diastolic relaxation [34, 35]. In contrast to volumetric assessment and even peak strain measures, strain rate analysis is not only based on quantification of end-diastolic and systolic frames but relies on the rate of changes in myocardial deformation over time. Thus, it is logical that strain rate might be most impacted by INTP_TR_, which was also the case in our results, where strain rate clearly differed between non-INTP_TR_ and INTP_TR_. In fact, we found that especially for the RV in the longitudinal orientation, diastolic strain rates were rarely useable with the INTP_TR_. It is important to mention that these were quantification errors and not tracking errors. In all images the readers determined adequate tracking by observing the displays of the software. The image exclusion occurred at the interrogation of the strain rate curves (i.e. it can be visualized in Fig. 6, the strain rate curve in it is unreliable). Consequently, supporting our recommendation stated above, if strain rates are desired by the analyser the non-INTP_TR_ should be quantified.

### Circumferential or longitudinal orientation

For both strain and strain rate, there was generally a better inclusion rate and agreement of measurements between INTP_TR_ and non-INTP_TR_ with the circumferential orientation, which can be observed in Figs. 2 and 3. These findings match trends reported for inter-reader reliability of strain analysis on standard cines where better agreement is observed for circumferential strain parameters than longitudinal [22, 36]. It is known that inter-reader reliability of feature tracking measurements is improved with a higher number of slices in the measurement [22], which could be a possible explanation for this finding as circumferential strain is typically analysed on a full short-axis stack which includes more slices than typical long-axis acquisitions. Especially for the RV in which longitudinal strain is analysed on a single slice, agreement and correlations of circumferential measurements were better, matching findings observed with LV circumferential measures.

### Should strain be improved by a faster temporal resolution?

With multiple imaging modalities it is described that with poorer (i.e. longer) TR, strain could be underestimated [37]. However, when images are acquired over multiple cardiac cycles, a shorter TR can be predefined at acquisition, and these non-real-time sequences aren’t reliant on retrospective TR shortening. With standard imaging, shorter time between images should theoretically improve accuracy as rapid movement especially in higher heart rates would be detected. However, with INTP_TR_ there is likely an optimal TR range. While the INTP_TR_ improves TR and provides equal phase counts between cines with different heart rates, it can introduce temporal blurring [38]. This is likely why FT strain was particularly impacted in comparison to anatomical assessments, as the algorithm relies on following specific features. Thus, we can interpret that the underestimation by FT is more compromised by the reconstruction process compared to the benefits that may arise from a shorter TR. In particular, the strain rates were more dependent on the TR, as a larger discrepancy was observed when the interpolated TR was significantly shorter than the preset non-interpolated TR. Consequently, if quantifying systolic or diastolic strain rates on INTP_TR_ images, values would be confounded by the RR-interval at the time of acquisition rather than the cardiac function. Conversely, peak strain values were not affected by the TR-ratio, indicating that one could have greater confidence in the output from INTP_TR_, even if the TR was significantly shortened.

### Limitations

Importantly, it should be noted that with the current version of the software used for analysis, assessment of the non-INTP_TR_ cines required more steps if the phase number differed, prolonging analysis time. Moreover, myocardial strain measures can differ by choice of software vendor as well, however for global analysis, and especially GCS, this difference can be limited [39, 40]. As multiple datasets were acquired per participant due to acquisitions at different levels, there remains a concern for oversampling from individual participants. Multiple comparisons per patient were not accounted for in the statistical models due to the smaller sample size. In particular, the subgroup analysis is limited with fourteen and nine datasets available for the analysis under a stimulus and under general anaesthesia, respectively. Future studies should interrogate larger sample sizes, especially to focus on differences that may occur under stress protocols with increased patient movement and heart rate. The primary goal was to assess if INTP_TR_ differed from non-INTP_TR_ and thus not as many datasets had a segmented cine acquisition for the comparisons to a gold-standard, as the segmented cines were primarily acquired in resting conditions and not under general anaesthesia or adenosine stress. In particular for the latter condition, further investigation could interrogate the role high heart rate may play when comparing CS cines either with or without INTP_TR_ to standard segmented protocols, which may be of greater relevance during stress or exercise protocols. Thus, the correlations and ROC comparisons to the segmented cine should be interpreted with caution due to the smaller sample size.

## Conclusion

As CS imaging integrates itself into mainstream imaging protocols, it is important to understand how features on CS sequences such as retrospective interpolation of temporal resolution impact the analysis of these images. Our analysis shows that readers can quantify standard biventricular and volume analysis on either output. If peak strain and strain rate measurements are desired, then analysis should be conducted on the non-INTP_TR_ CS datasets. This study successfully acquired CS imaging in a range of different haemodynamic conditions. Further investigation, quantifying cardiac function with strain assessment on non-INTP_TR_ CS cines is warranted, and may play an important role in understanding cardiac function in patients that could not be adequately imaged with traditional techniques.

## Electronic supplementary material

Below is the link to the electronic supplementary material.

Supplementary Material 1

## Data Availability

Data available on request due to privacy/ethical restrictions.
