# Supplementary material for: Retrospective temporal resolution interpolation alters myocardial strain quantification on compressed sensing cine CMR
Source: Int J Cardiovasc Imaging. 2025 Feb 14;41(3):591–602. doi: 10.1007/s10554-025-03348-3 (PMC11880142; doi:10.1007/s10554-025-03348-3)
Supplement: Supplementary file 1 — Supplementary Material 1 [file 10554_2025_3348_MOESM1_ESM.pdf]

SUPPLEMENTAL MATERIAL

Retrospective Temporal Resolution Interpolation Alters Myocardial Strain Quantification on Compressed Sensing Cine CMR

Table of Contents

SUPPLEMENTAL TABLE 1: BIVENTRICULAR VOLUMES AND MASS..2

SUPPLEMENTAL TABLE 2: STRAIN MEASURES 3

SUPPLEMENTAL TABLE 3: CORRELATION COEFFICIENTS...4

SUPPLEMENTAL TABLE 4: CORRELATION OF THE DIFFERENCE IN NON-INTP$_{TR}$ TO INTP$_{TR}$ IN COMPARISON TO THE TR RATIO 5

SUPPLEMENTAL TABLE 5: AUC MEASURES..6

SUPPLEMENTAL TABLE 6: CORRELATION OF THE DIFFERENCE IN INTP$_{TR}$ AND NON-INTP$_{TR}$ TO STANDARD SEGMENTED IN COMPARISON TO HEART RATE AT THE TIME OF ACQUISITION 7

Supplemental Table 1: Biventricular volumes and mass

Function	$INTP_{TR}$	n	Non-$INTP_{TR}$	n
Left Ventricle				
End Diastolic $Volume_{index}$ (ml/m^2)	74±12	49	74±11	49
>96 (ml/m^2) for females<br>>108 (ml/m^2) for males	0 (0)		0 (0)	
End Systolic $Volume_{index}$ (ml/m^2)	32±6	49	32±6	49
Ejection Fraction (%)	56±4	49	57±5	49
<45% (n,%)	0 (0)		0 (0)	
Cardiac Index (l/min/m^2)	2.9±0.7	49	2.9±0.7	49
Mass Index (g/m^2)	51±9	49	52±9	49
>68 (g/m^2) for females<br>>85 (g/m^2) for males	0 (0)		0 (0)	
Right Ventricle				
End Diastolic $Volume_{index}$ (ml/m^2)	91±19	49	90±16	49
End Systolic $Volume_{index}$ (ml/m^2)	50±14	49	49±13	49
Ejection Fraction (%)	45±8	49	46±8	49
<40% (n,%)<br><30% (n,%)	16 (33)<br>0 (0)		13 (27)<br>0 (0)	
Cardiac Index (l/min/m^2)	2.9±0.9	49	2.9±0.9	49

Mean±SD, along with sample size are shown for functional measurements from $INTP_{TR}$, non-$INTP_{TR}$ and segmented sequences. For some key functional measurements, datasets were stratified if they outside of common reference ranges. End diastolic volume and end systolic volume are indexed to body surface area. $INTP_{TR}$ = retrospective temporal resolution interpolation.

Supplemental Table 2: Strain Measures

Strain	$INTP_{TR}$	n	Non-$INTP_{TR}$	n	Segmented	n
Left Ventricle						
Peak Strain (%)						
Circumferential	-15.6±2.0	48	-17.3±1.9	49	-18.7±2.9	17
Longitudinal	-12.6±2.4	37	-15.3±2.4	37	-17.7±2.0	17
Systolic Strain Rate (/s)						
Circumferential	-0.97±0.25	47	-0.96±0.15	49	-0.86±0.15	17
Longitudinal	-0.83±0.18	22	-0.85±0.18	36	-0.84±0.09	17
Early Diastolic Strain Rate (/s)						
Circumferential	0.83±0.33	46	1.04±0.34	48	0.81±0.19	17
Longitudinal	0.63±0.18	22	0.86±0.30	35	0.81±0.22	17
Late Diastolic Strain Rate (/s)						
Circumferential	0.35±0.15	36	0.35±0.13	33	0.58±0.14	17
Longitudinal	0.30±0.11	17	0.42±0.17	31	0.81±0.17	17
Right Ventricle						
Peak Strain (%)						
Circumferential	-10.1±3.0	47	-11.1±3.6	49	-12.8±4.6	17
Longitudinal	-17.2±4.8	33	-20.8±3.1	33	-24.5±3.2	17
Systolic Strain Rate (/s)						
Circumferential	-0.61±0.20	38	-0.64±0.30	49	-0.72±0.27	17
Longitudinal	-1.40±0.50	7	-1.30±0.35	32	-1.20±0.36	17
Early Diastolic Strain Rate (/s)						
Circumferential	0.53±0.21	37	0.61±0.20	49	0.55±0.18	17
Longitudinal	1.12±0.94	7	0.98±0.40	32	0.77±0.23	17
Late Diastolic Strain Rate (/s)						
Circumferential	0.26±0.14	21	0.28±0.18	36	0.33±0.20	17
Longitudinal	0.59±0.57	5	0.76±0.33	27	1.34±0.47	17

Mean±SD, along with sample size are shown for strain measurements from $INTP_{TR}$, non-$INTP_{TR}$ and segmented sequences.

Supplemental Table 3: Correlation Coefficients

	Non-$INTP_{TR}$ vs $INTP_{TR}$ r	Non-$INTP_{TR}$ vs $INTP_{TR}$ p	Non-$INTP_{TR}$ vs Segmented r	Non-$INTP_{TR}$ vs Segmented p	$INTP_{TR}$ vs Segmented r	$INTP_{TR}$ vs Segmented p
Left Ventricle						
Volumetry						
End Diastolic $Volume_{index}$ (ml/m^2)	0.99	<0.01	0.98	<0.01	0.99	<0.01
Ejection Fraction (%)	0.94	<0.01	0.88	<0.01	0.90	<0.01
Peak Strain (%)						
Circumferential	0.81	<0.01	0.82	<0.01	0.69	<0.01
Longitudinal	0.66	<0.01	0.83	<0.01	0.88	<0.01
Systolic Strain Rate (/s)						
Circumferential	0.71	<0.01	0.56	0.03	0.68	<0.01
Longitudinal	0.43	0.89	0.57	0.33	-	-
Early Diastolic Strain Rate (/s)						
Circumferential	0.75	<0.01	0.78	<0.01	0.67	0.01
Longitudinal	0.44	0.88	0.78	0.01	-	-
Late Diastolic Strain Rate (/s)						
Circumferential	0.03	0.05	0.76	<0.01	0.77	<0.01
Longitudinal	-	-	0.58	0.13	-	-
Right Ventricle						
Volumetry						
End Diastolic $Volume_{index}$ (ml/m^2)	0.95	<0.01	-	-	-	-
Ejection Fraction (%)	0.95	<0.01	-	-	-	-
Peak Strain (%)						
Circumferential	0.90	<0.01	0.91	<0.01	0.88	<0.01
Longitudinal	0.66	<0.01	0.87	0.03	0.64	0.17
Systolic Strain Rate (/s)						
Circumferential	0.89	<0.01	0.79	<0.01	0.65	0.02
Longitudinal	-	-	0.63	0.19	-	-
Early Diastolic Strain Rate (/s)						
Circumferential	0.57	<0.01	0.22	0.43	0.11	0.73
Longitudinal	-	-	-0.75	0.09	-	-
Late Diastolic Strain Rate (/s)						
Circumferential	0.46	0.05	0.52	0.07	0.77	0.03
Longitudinal	-	-	0.61	0.27	-	-

Pearson's correlation coefficients along with p-values are shown. If comparisons had less than 10 measurements, they were not compared. $INTP_{TR}$ = retrospective temporal resolution interpolation.

Supplemental Table 4: Correlation of the difference in non-INTP$_{TR}$ to INTP$_{TR}$ in comparison to the TR Ratio

	Left Ventricle		Right Ventricle	
	r	p	r	p
**Volumes**				
End Diastolic Volume$_{index}$	-0.27	0.07	-0.30	0.04
Ejection Fraction (%)	-0.19	0.20	-0.10	0.49
**Feature Tracking Strain**				
Peak Strain (%)				
Circumferential	-0.27	0.07	0.00	0.99
Longitudinal	0.04	0.81	0.00	0.97
Systolic Strain Rate (/s)				
Circumferential	0.77	<0.01	-0.27	0.17
Longitudinal	0.43	0.05	0.70	0.08
Early Diastolic Strain Rate (/s)				
Circumferential	0.25	0.09	-	-
Longitudinal	-0.07	0.24	-	-

Pearson's correlation coefficients along with p-values are shown comparing the difference in the measurements between the non-INTP$_{TR}$ and INTP$_{TR}$ and the ratio of the TR ratio (TR of the non-INTP$_{TR}$/INTPTR) between the outputs. If comparisons had less than 10 measurements, they were not compared. INTP$_{TR}$ = retrospective temporal resolution interpolation.

Supplemental Table 5: AUC Measures

		Non-$INTP_{TR}$		$INTP_{TR}$	
	Segmented Cine Cutoff	AUC	p	AUC	p
**Left Ventricle**					
GCS	> -16.9%	0.98	<0.01	0.95	<0.01
Systolic SR_{circ}	> -0.80/s	0.94	<0.01	0.83	0.04
Early Diastolic SR_{circ}	< 0.92/s	0.91	<0.01	0.80	0.08
GLS	> -16.5%	1.00	<0.01	1.00	<0.01
Systolic SR_{long}	> -0.71/s	0.76	0.21	-	-
Early Diastolic SR_{long}	< 0.90/s	0.93	0.07	-	-

Area under the curve (AUC) and p-values depict the diagnostic capability of the measurements from the compressed sensing cines in comparison to patients with abnormal strain measurements on the standard segmented cine. The cut-off to define abnormal patients from the segmented cine was obtained from normal site values. If comparisons had less than 10 measurements, they were not compared. $INTP_{TR}$ = retrospective temporal resolution interpolation.

Supplemental Table 6: Correlation of the difference in $INTP_{TR}$ and non-$INTP_{TR}$ to standard segmented in comparison to heart rate at the time of acquisition

	**Non-$INTP_{TR}$**		**$INTP_{TR}$**	
	**r**	**p**	**r**	**p**
**Feature Tracking Strain**				
Peak Strain (%)				
Circumferential	-0.35	0.20	-0.33	0.24
Longitudinal	-0.46	0.25	0.26	0.54
Early Diastolic Strain Rate (/s)				
Circumferential	-0.05	0.86	0.27	0.37
Longitudinal	-	-	-	-

Pearson's correlation coefficients along with p-values are shown show that the difference in the measurements between the $INTP_{TR}$/non-$INTP_{TR}$ and standard segmented cines was not related to the heart rate at the time of acquisition which ranged from 46-74bpm. If comparisons had less than 10 measurements, they were not compared. These comparisons do not include data obtained under adenosine or general anaesthesia as segmented standard cines were not acquired at these levels.
$INTP_{TR}$ = retrospective temporal resolution interpolation.
